# Expiratory phase lung mechanics in late-onset Pompe disease: a multicenter study using oscillometry to identify specific breathing abnormalities

**DOI:** 10.1186/s13023-026-04532-x

**Published:** 2026-08-07

**Authors:** Grazia Crescimanno, Oreste Marrone, Marta Lazzeri, Paolo Innocente Banfi, Agata Lax, Elena Compalati, Rosario Di Marco, Sabrina Planiscig, Paola Confalonieri, Fabrizio Seidita, Filippo Brighina, Marco Confalonieri

**Affiliations:** 1 Institute for Biomedical Research and Innovation (IRIB), National Research Council, Palermo, Italy; 2 Departmental Unit of Neurophysiopathology, Regional Centre for Diagnosis and Treatment of Rare Neurological Diseases, AOUP Policlinico, University of Palermo, Palermo, Italy; 3 IRCCS Fondazione Don Gnocchi, Milano, Italy; 4 Department of Pulmonology, University Hospital of Cattinara, Trieste, Italy; 5 Functional Recovery and Rehabilitation, Ospedali Riuniti Villa Sofia- Cervello, Palermo, Italy; 6 AIGlico (Italian Glycogenosis Association), Assago, Italy

**Keywords:** Late-onset Pompe disease, Respiratory oscillometry, Intrabreath analysis, Respiratory impedance, Air-stacking

## Abstract

**Introduction:**

In late-onset Pompe disease (LOPD), muscle weakness causes restrictive lung impairment. In murine models, glycogen deposition in lung parenchyma suggests additional causes for restriction that remain poorly understood. We investigated whether oscillometry detects respiratory abnormalities not identified by spirometry and evaluated changes after air-stacking manoeuvres.

**Methods:**

We prospectively evaluated 16 adults with LOPD from three Italian centres. Alongside spirometry, oscillometry (at 5, 11, and 19 Hz) was performed to assess resistance (R) and reactance (X) across the breathing cycle and by respiratory phase. Measurements were repeated 5 min after an air-stacking manoeuvre.

**Results:**

Participants (7 men, 9 women; 51.6 ± 12.5 years) had moderate-to-severe restriction (FVC 53.3 ± 19.6% predicted) with preserved FEV1/FVC. Two oscillometric patterns emerged. The first one (seven patients) was characterised by an abnormal increase in expiratory resistance within breaths ([R5exp − R5insp] > 0.70 cmH_2_O·s/L), associated or not with abnormal whole-breath R and intrabreath X changes. The second pattern (nine patients) consisted of normal R and X changes in the breathing cycle. Air-stacking increased FVC (53.3 ± 19.6 to 71.3 ± 19.2% predicted, *p* < 0.001) without normalising reactance.

**Conclusion:**

In LOPD, oscillometry detects distinct mechanical patterns not identified by spirometry. Air-stacking increases lung volumes without improving respiratory mechanics.

## Letter to the Editor

Late-onset Pompe disease (LOPD) is a rare lysosomal metabolic myopathy in which diaphragmatic weakness leads to restrictive ventilatory impairment and influences clinical outcomes [1]. Although respiratory failure is typically attributed to neuromuscular weakness, experimental models of acid alpha-glucosidase deficiency show glycogen accumulation in distal lung structures and airways, suggesting possible non-muscular pulmonary involvement [2].

Lung-volume recruitment manoeuvres, such as air-stacking, are widely used to transiently increase lung volume in neuromyopathies [3]. The forced oscillation technique (FOT) provides effort-independent assessment of respiratory impedance, distinguishing airway resistance (R) from reactance (X) [4].

We aimed to evaluate whether FOT can identify respiratory abnormalities in LOPD patients with extraparenchymal restrictive impairment not detected by spirometry and to assess the effects of air-stacking on phase-specific airway mechanics.

This was a prospective observational study of 16 adults with confirmed LOPD and respiratory involvement (FVC < 80% predicted or postural FVC fall > 25%), recruited across three Italian centres. Ethics approval was obtained locally, and all participants provided written informed consent. Spirometry and maximal inspiratory/expiratory pressures (MIP/MEP) were obtained according to ATS/ERS standards; arterial blood gases were measured at rest [5–7]. FOT was performed with a device compliant with European Respiratory Society recommendations (RESMON PRO FULL, Restech srl, Milan, Italy) at 5, 11 and 19 Hz. Oscillometry measurements were standardised across participating centres using the same device and acquisition protocol and were performed in the sitting position in all participants. Whole-breath and phase-specific indices were considered abnormal based on z-score thresholds [8]. R5 measures resistance at 5 Hz (sensitive to total airway resistance), while X5 measures reactance at 5 Hz (sensitive to elastic properties of the peripheral airways). To evaluate the effects of air stacking, X11 was selected instead of X5 because reactance at higher frequencies may be less affected by peripheral airway heterogeneity. Therefore, it may provide a more stable representation of global changes in respiratory system mechanics associated with lung volume recruitment. Intrabreath indices were defined as ΔR5 = R5exp − R5insp and ΔX5 = X5exp − X5insp.

Predicted intrabreath expiratory-inspiratory differences are small in healthy adults [9]. We wanted to adopt highly specific thresholds to unequivocally identify abnormal intrabreath oscillometric differences. For that purpose, we used threshold values > 0.70 cmH_2_O·s/L for ΔR5, and < -1.0 cmH_2_O·s/L for ΔX5, which are greater than those established with reference equations for the general population [9]. A high ΔR reflects a heterogeneous increase in expiratory airway resistance, while a highly negative ΔX indicates increased mechanical instability during expiration, reflecting greater heterogeneity in respiratory system mechanics, or dynamic airway closure.

Measurements were accepted only if artefact-free and repeatable. If whole-breath R5 exceeded the upper normal limit, a bronchodilator test was performed on a different day [10]. After baseline oscillometry, all patients performed a standardised air-stacking manoeuvre under the supervision of a respiratory therapist. Consecutive insufflations were delivered using a manual resuscitation bag until maximal insufflation capacity was achieved and recorded. FOT and spirometry were repeated 5 min after the manoeuvre.

Wilcoxon or t test were used for paired comparisons as appropriate; between-group comparisons were tested by Mann-Whitney or t tests; correlations were explored with Spearman’s rho (two-sided *p* < 0.05). Potential confounding effects of age, sex, BMI, functional status, and disease duration were explored in groups defined by oscillometric indices.

Baseline characteristics are summarised in Table 1. Participants showed moderate-to-severe restriction (FVC 53.3 ± 19.6% predicted) with preserved Tiffenau index (FEV1/FVC 82.7 ± 10.9%). No one had a history of smoking (except one e-cigarette user) or pre-existing respiratory comorbidities. Bronchodilator testing, performed in three participants, showed no response. None of the included patients had a clinical or radiological diagnosis of interstitial lung disease. Available chest CT examinations did not demonstrate diffuse parenchymal abnormalities suggestive of intrinsic restrictive lung disease.

**Table 1:** Clinical, respiratory function and oscillometric characteristics by intrabreath expiratory limitation pattern

Clinical variables and respiratory function			
	expiratory limitation (# 7)	without expiratory limitation (9)	*p* value
Age (mean ± SD)	55.2 ± 9.5	48.8 ± 14.2	0.310
Sex (# F/M, Females/Males)	5 F/2 M	4 F/5 M	0.290
BMI (kg/m2) (mean, ± SD)	25.6 ± 5.3	22.1 ± 2.5	0.110
Functional status	6 walking	8 walking	0.140
NIV (patients, %)	5 NIV (71.4%)	7 NIV (77.7%)	0.770
Years of disease (mean ± SD)	17.2 ± 4.0	17.5 ± 5.5	0.940
FVC L	2.06 ± 0.5	1.89 ± 1.2	0.76
FVC%	57.0 ± 8.2	48.1 ± 26.2	0.40
FEV1 L	1.7 ± 0.5	1.5 ± 1.1	0.72
FEV1%	57.4 ± 11.0	47.7 ± 24.4	0.35
FEV1/FVC %	83.4 ± 9.4	84.3 ± 11.6	0.86
MIP cmH_2_O	-54.7 ± 12.8	-35.4 ± 20.5	0.04
MEP cm H_2_O	70.4 ± 19.9	43.6 ± 13.6	0.001
**Whole breath oscillometry**			
R5 cmH₂O·s/L (mean, SD)	4.6 [2.8 to 5.0]	1.8 [1.6 to 2.0]	0.0039
R11 cmH₂O·s/L(mean, SD)	4.4 [2.9 to 4.8]	1.8 [1.6 to 2.1]	0.0036
R19 cmH₂O·s/L (mean, SD)	4.1 [2.7 to 4.2]	1.7 [1.5 to 2.1]	0.0058
R5-19 (median, IQR)	0.42 [0.12 to 0.73]	-0.06 [-0.30 to 0.26]	0.039
X5 cmH₂O·s/L (median, IQR)	-1.28 [-2.1to -1.0]	-0.74 [-1.0 to -0.3]	0.026
X11 cmH₂O·s/L (median, IQR)	-0.43 [-1.3 to -0.3]	0.28 [-0.3 to 0.8]	0.017
X19 cmH₂O·s/L(median, IQR)	-0.05 [-0.19 to 0.5]	1.0 [0.7 to 1.3]	0.003
FRES (Hz) * (median, IQR)	14.4 [11.9 to 18.8]	9.9 [7.7 to 11.7]	0.054
AX cmH₂O·s/L * (median, IQR)	3.8 [2.3 to 7.8]	2.2 [0.6 to 2.6]	0.200
**Intrabreath oscillometry**			
R5 inspiratory/expiratory (mean, SD)	3.4 ± 1.2/4.7 ± 1.5	1.8 ± 0.5/2.0 ± 0.5	
ΔR5 (median, IQR)	1.0 [0.7 to 1.8]	0.09 [0.03 to 0.30]	0.0008
R11inspiratory/expiratory (mean, SD)	3.3 ± 1.0/4.5 ± 1.2	1.9 ± 0.6/2.1 ± 0.6	
Δ R11 (median, IQR)	1.0 [0.7 to 1.4]	0.2 [0.04 to 0.3]	0.0012
R19 inspiratory/exp (mean, SD)	3.2 ± 0.9/4.0 ± 1.2	1.7 ± 0.6/2.0 ± 0.5	
Δ R19 (median, IQR)	0.86 [0.3 to 1.3]	0.24 [0.1 to 0.3]	0.039
X5inspiratory/expiratory (mean, SD)	-1.0 ± 0.4/-1.9 ± 0.9	-0.7 ± 0.6/-0.7 ± 0.7	
Δ X5 (median, IQR)	-0.48 [-1.5 to-0.3]	0.03 [-0.03 to 0.1]	0.0042
X11 inspiratory/expiratory (mean, SD)	-0.39 ± 0.4/-1.0 ± 0.8	0.04 ± 0.5/0.02 ± 0.5	
ΔX11 (median, IQR)	-0.30 [-1.2 to -0.17]	-0.02 [-0.08 to 0.07]	0.0041
X19 inspiratory/expiratory (mean, SD)	0.4 ± 0.3/-0.18 ± 0.6	0.9 ± 0.4/0.98 ± 0.5	
ΔX19 (median, IQR)	-0.35 [-0.8 to -0.2]	0.01 [-0.03 to 0.04]	0.0008

*FRES: resonant frequency; AX: reactance area. Intrabreath oscillometric variables are presented as inspiratory/expiratory values (mean ± SD). Δ values represent expiratory − inspiratory differences and are reported as median [IQR]. p-values refer to between-group comparisons of Δ-values. Units for intrabreath oscillometric parameters are the same as those reported for corresponding whole-breath variables. Data are reported as absolute values. Analyses performed using z-scores yielded similar results (data not shown)

### All patients were receiving enzyme replacement therapy

**Fig. 1:**
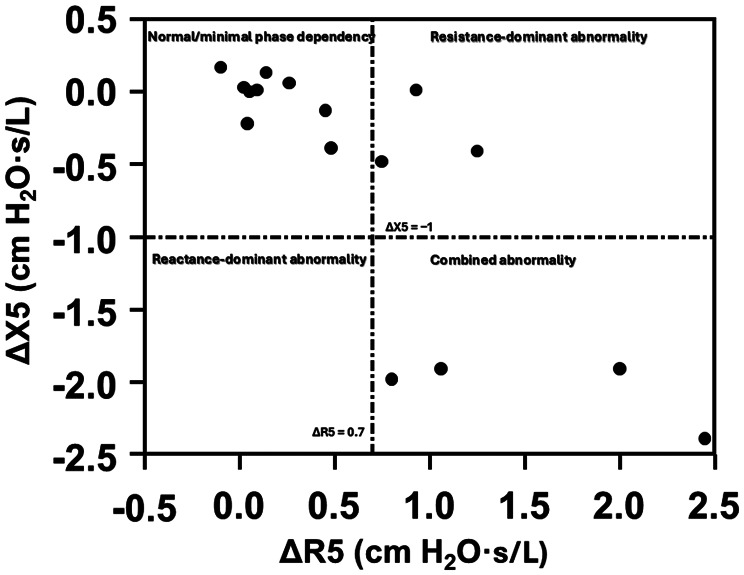
Intrabreath resistance–reactance quadrant plot. Scatter plot showing intrabreath changes in resistance (ΔR5) and reactance (ΔX5) during tidal breathing. Each point represents one patient. Vertical and horizontal lines indicate the predefined thresholds used to identify expiratory mechanical instability (ΔR5 > 0.70 cmH₂O·s/Land ΔX5 < − 1 cmH₂O·s/L). The lower-right quadrant corresponds to patients fulfilling both criteria, consistent with expiratory airway instability. The upper-right quadrant represents patients with increased expiratory resistance without deterioration in reactance, while the upper-left quadrant includes mechanically stable patients. No patients were observed in the quadrant characterised by isolated deterioration in reactance (ΔX5 < − 1 without increased ΔR5)

Two oscillometric patterns were identified. The first pattern was observed in seven patients who met the prespecified ΔR5 threshold for a heterogeneous increase in expiratory airway resistance. Among them, four had normal whole-breath R5, and three had elevated R5, indicating that abnormal expiratory mechanics can occur despite normal overall resistance. Four of the seven subjects also showed marked expiratory reactance abnormality. The second pattern was found in the remaining nine patients who maintained normal intrabreath R and X throughout the breathing cycle (Fig. 1).

Patients without expiratory-phase abnormality had significantly lower respiratory muscle strength despite similar spirometry and blood gas values.

Notably, neither ΔR5 nor ΔX5 was correlated with baseline FVC% (rho = -0.08, *p* = 0.70; rho = 0.08, *p* = 0.75, respectively), suggesting that lung volume reduction alone could not explain the observed mechanical abnormalities.

**Fig. 2:**
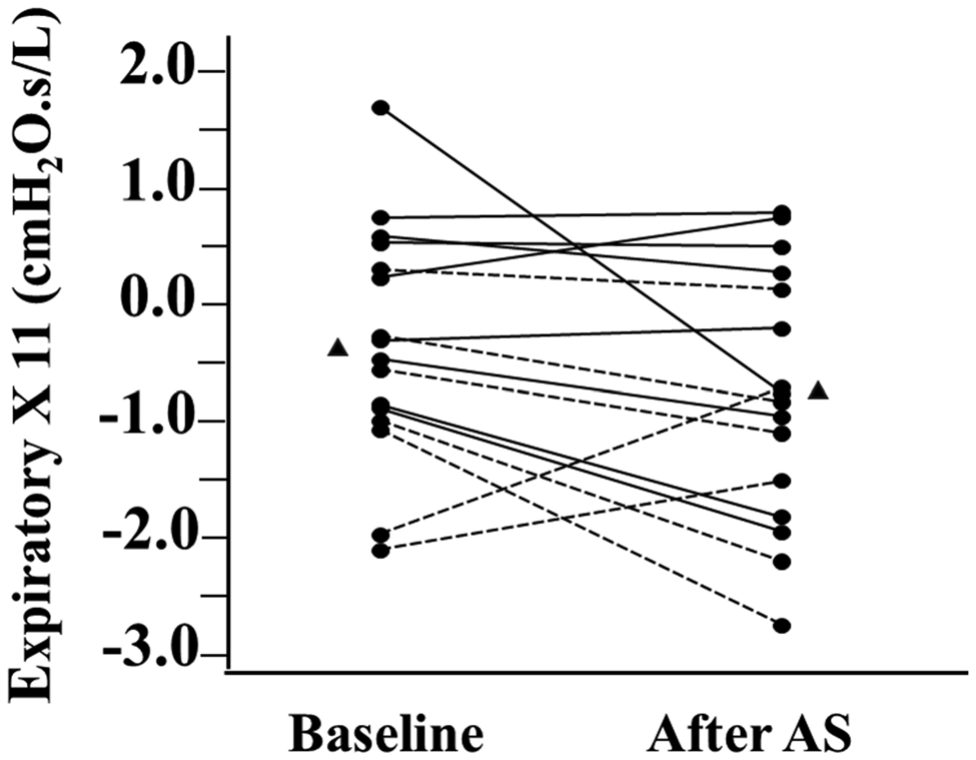
Expiratory Reactance at 11 Hz (X11) before and after air-stacking,. ---- = Pts with expiratory limitation; –– = Pts without expiratory limitation; = median

Air-stacking produced a clear immediate increase in lung volume, reflected by improved FVC (53.3 ± 19.6 to 71.3 ± 19.2% predicted; *p* < 0.001). However, X11 did not change significantly following air-stacking. Instead, a trend toward worsening was observed, despite marked interindividual variability, as shown in Fig. 2.

This prospective multicentre series of adults with LOPD and clinically relevant respiratory involvement shows that, in some patients, intrabreath oscillometry identifies expiratory-phase mechanical abnormalities not captured by spirometry or whole-breath resistance alone. Two clinically plausible respiratory mechanical profiles emerged: (i) an ‘expiratory-phase abnormality’ pattern, in which intrabreath indices suggest airway–parenchymal mechanical involvement alongside global restriction; and (ii) a pattern with normal intrabreath indices, more consistent with predominant neuromuscular restriction without detectable expiratory-phase instability at rest.

The biological basis for heterogeneous increase in expiratory resistance remains uncertain. Although respiratory muscle weakness is central to LOPD, experimental data indicate that glycogen accumulation may involve distal airway and peripheral lung structures, potentially contributing to altered regional mechanics [2]. In our cohort, lower expiratory muscle strength among patients without expiratory-phase abnormality may reduce expiratory driving pressure, thereby attenuating the oscillometric expression of dynamic narrowing; however, this interpretation is speculative and requires dedicated physiological validation. A larger sample size could provide stronger evidence of the relationship between respiratory muscle strength and intrabreath oscillometric findings, which, at present, should be regarded with caution.

Air-stacking produced a clear, immediate increase in lung volume, as reflected by an improvement in FVC. In contrast, X11 did not show a uniform direction of change. These findings may suggest that acute lung volume recruitment does not necessarily result in uniform modifications in intrabreath respiratory mechanics. Possibly, in some patients, it is associated with changes in airway–parenchyma interactions or expiratory mechanical heterogeneity. Importantly, our observations should not be interpreted as evidence against lung-volume recruitment therapy.

From a clinical perspective, intrabreath oscillometry could complement standard assessments in LOPD [5] by providing a noninvasive, effort-independent signal of expiratory-phase mechanics, potentially helping to refine phenotyping and longitudinal monitoring. Whether the identified patterns predict symptoms, response to ventilatory support, mucus clearance efficacy, or clinical outcomes should be tested in larger prospective cohorts. Longitudinal studies would be appropriate to separately evaluate long-term evolution of clinical features in LOPD patients with and without expiratory-phase oscillometric abnormalities.

Our study is limited by a small sample size, the absence of a control groupand the presence of potential unmeasured confounders, including differences in enzyme replacement therapy. DLCO assessment was not routinely performed. In addition, thresholds for abnormal oscillometric intrabreath measures have not been specifically validated in LOPD or neuromuscular populations and should therefore be considered exploratory rather than disease-specific diagnostic criteria. Although oscillometry does not localise the underlying pathology, it provides clinically relevant information beyond spirometry.

In conclusion, intrabreath oscillometry identified two distinct airway-mechanical patterns and expiratory abnormalities not detected by spirometry. An abnormal expiratory-phase pattern may reflect greater clinical respiratory severity despite similar spirometric impairment. Given the small number of patients, larger studies are needed to determine whether these patterns reflect distinct disease phenotypes. Air-stacking increased FVC but did not consistently normalise intrabreath mechanics, underscoring the potential multifactorial nature of respiratory dysfunction in LOPD.

## Data Availability

The data that support the findings of this study are available from the corresponding author upon reasonable request. Declarations.

## Declarations

### Notation of prior abstract publication/presentation

This study represents original research and has not been previously presented in any form, including as an abstract or oral presentation.

### “Take home” message

Oscillometry provides a non-invasive, effort-independent evaluation of expiratory-phase mechanics, with the potential to improve characterization and long-term monitoring of respiratory involvement in LOPD patients.

## Abbreviations

FVC: Forced vital capacity
FEV1: Forced expiratory volume in 1 s
FEV1/FVC: Tiffenau index
FOT: Forced oscillation technique
R5: Resistance at 5 Hz
R11: Resistance at 11 Hz
R19: Resistance at 19 Hz
X5: Reactance at 5 Hz
X11: Reactance at 11 Hz
ΔR5: Intrabreath difference in R5 (expiratory − inspiratory)
ΔX5: Intrabreath difference in X5 (expiratory − inspiratory)
ΔX11: Intrabreath difference in X11 (expiratory − inspiratory)
